# Development of multi-layered and multi-sensitive polymeric nanocontainers for cancer therapy: in vitro evaluation

**DOI:** 10.1038/s41598-018-32890-5

**Published:** 2018-10-02

**Authors:** Gianluca Toniolo, Eleni K. Efthimiadou, George Kordas, Chryssostomos Chatgilialoglu

**Affiliations:** 1 Institute of Nanoscience and Nanotechnology, NCSR Demokritos, Athens, Greece; 2 Institute for Organic Synthesis and Photoreactivity ISOF, Italian National Research Council, Bologna, Italy; 3 Inorganic Chemistry Laboratory, Chemistry Department, National and Kapodistrian University of Athens, Panepistimiopolis, Zografou, Greece

## Abstract

Nanoscale drug delivery systems represent a promising strategy to treat cancer and to overcome the side effects of chemotherapy. In particular, hollow polymeric nanocontainers have attracted great interest because of their structural and morphological advantages and the variety of polymers that can be used, allowing the synthesis of stimuli-responsive materials capable of responding to the biochemical alterations of the tumour microenvironment. Here are reported the synthesis, characterization and *in vitro* evaluation of a three-stimuli-sensitive hollow nanocontainer consisting of three different shells, each one sensitive to a specific tumoral stimulus: in order pH, temperature and reducing environment. To test its properties, daunorubicin was used as a model drug, for which the nanocontainers exhibited excellent encapsulation ability. The *in vitro* drug release behaviour was studied under different conditions, where the system proved capable of responding to the selected tumoral stimuli by releasing a larger amount of drug than in physiological environment. The hollow system itself showed negligible cytotoxicity but the loaded nanocontainers and free drug showed identical cytotoxicity and intracellular localization. Therefore, this formulation can be considered as a promising platform to develop an injectable delivery system capable of improving systematic toxicity without affecting or reducing the activity of the encapsulated drug.

## Introduction

Conventional chemotherapeutic agents lack in specificity and selectivity, which is too often translated into unpleasant side effects for the patient. Since their ideation, nanoscale drug delivery systems have proposed a revolutionary way to treat cancer and to overcome the toxicity problems associated to the current therapies^1–3^. These systems are capable of specifically targeting tumoral tissues because of their size, which permits to exploit the enhanced penetration and retention (EPR) effect^4^. This is a characteristic phenomenon of solid tumours, based on their anatomical and pathophysiological differences from normal tissues. In particular, the overstimulated and defective angiogenesis of tumours causes large gaps in the new vessels, which result leaky. A drug delivery system with a compatible size can take advantage of this and selectively enter into the unhealthy tissue. In addition, the inefficient lymphatic drainage guarantees the retention of the delivery agents in the area, permitting the accumulation of the drug^5,6^.

Recently, many multistimuli-sensitive or “smart” drug delivery systems have been proposed. Indeed, tumoral cells and tissues are characterized by some internal biochemical alterations, that can serve as a specific trigger for facilitating the release of the loaded drug^7,8^. Among these alterations, the best known and most exploited are (i) the lower pH compared to normal tissues^9–11^, (ii) the concentration of glutathione^12–14^, which is at least four-fold higher in tumoral cells^15^ and (iii) the difference in temperature^16–18^, higher in tumoral areas but also increasable with external sources (e.g. hyperthermia treatments)^19,20^. Lately, some multi-stimuli-sensitive delivery systems, capable of responding to more than one stimulus, have been reported as well^21–25^.

Among all the available drug delivery systems, hollow polymeric nanocontainers (NCs) are considered one of the most promising options^26^. A proper design and choice of components allow the easy synthesis of different types of stimuli-responsive materials and, on the other hand, the central cavity can accommodate a much bigger amount of drug compared to the other systems^27^.

Here, we report the synthesis, characterization and property investigation of a three-stimuli-sensitive hollow NC, capable of responding to pH, temperature and redox variations. The system consists of three different shells obtained layer-by-layer via distillation-precipitation polymerization, and each of them is responsible for one specific sensitivity. Although drug delivery agents usually concentrate all the sensitivities in a single shell, we report a system made of three different shells, aiming at gaining more control on the sensitivities by optimizing each synthesis step, and therefore at being able to tune the properties both independently and in relation to one another. The first shell, pH sensitive, was synthesized by using methacrylic acid (MAA) as the main monomer. The carboxylic groups of the resulting PMAA shell can be protonated or deprotonated depending on the pH (pKa ca. 4.5), and therefore differently interact with the external environment^28,29^. The second shell is capable of responding to temperature variations because of N,N′-dimethylaminoethyl methacrylate (DMAEMA), the polymer of which is known to be temperature and pH responsive^30,31^. The outermost shell is redox sensitive, owing to N,N-Bis(acryloyl)cystamine (BAC), crosslinking agent with a disulphide bond and therefore easily breakable in reducing environments^15,20^. The *in vitro* drug release behaviour under the proposed stimuli was tested by using daunorubicin (DNR) as a model drug. DNR, among the most used chemotherapeutics, is classified as an anthracycline antitumor antibiotic with a pronounced impact on a broad range of tumours, but it is also responsible for cardiotoxicity and other severe side effects^32,33^. The new formulation was also tested and evaluated in terms of *in vitro* cytotoxicity and subcellular localization.

## Methods

### Materials

Methacrylic acid (MAA, 99%), 2,2′-Azobisisobutyronitrile (AIBN, 98%) and N,N′-methylenebisacrylamide (MBA, 96%) were purchased from Acros. N,N-dimetilaminoethylethacrylate (DMAEMA), Poly(ethyleneglycol)methylethermethacrylate (PEGMA; Mn = 475) and acetonitrile (ACN) were obtained from Sigma-Aldrich., N′-bis(acryloyl)cystamine (BAC, 98%) was purchased from Alfa Aesar. Daunorubicin HCl (DNR) was provided by Pharmacia & Upjohn.

### Equipment

Scanning electron microscopy (SEM) images were obtained with FEI Inspect microscope with W (tungsten) filament operating at 25 kV. Fourier transform infrared (FT-IR) spectra were obtained with Perkin- Elmer Spectrum 100 spectrometer. The dynamic light scattering (DLS) measurements were performed with a Malvern Instruments Zetasizer Nano Series, with a multipurpose titrator. In the data presented in this study, each measurement represents the average value of 5 measurements, with 11–15 runs for each measurement.

### Synthesis of PMAA cores

MAA (2.1 g, 24.4 mmol) was dissolved in 200 ml of ACN and let stir for 30 min at 75 °C under nitrogen bubbling before adding AIBN (0.3 g, 1.8 mmol). When the flask content turned milky, the temperature of the system was increased to 95 °C to start the distillation. After collecting 20 ml of ACN, the reaction was stopped and the product was purified with 3 cycles of centrifugation resuspension (5 min × 8000 rpm) in ACN and collected as a white powder.

### Synthesis of PMAA@P(MAA-co-PEGMA-co-MBA)

PMAA cores (0.15 g) were suspended with ultrasonic bathing in 200 ml of ACN. The suspension was maintained under agitation at 75 °C and nitrogen bubbling for 30 min prior to add the other components. MAA (0.53 g, 6.1 mmol) was added first, and after 10 min also PEGMA (0.16 g, 0.3 mmol) and MBA (0.15 g, 0.97 mmol). The medium was let stir for 30 and then AIBN (0,09 g, 0,5 mmol) was added. 10 min later, the temperature was increased to 95 °C to start the distillation. The reaction was stopped after collecting 30 ml of distilled ACN and the product was purified with 3 cycles of centrifugation resuspension in ACN (5 min × 10000 rpm) and collected as a white powder.

### Synthesis of PMAA@P(MAA-co-PEGMA-co-MBA)@P(DMAEMA-co-MAA-co-PEGMA-co-MBA)

PMAA@P(MAA-co-PEGMA-co-MBA) (0.2 g) were suspended in 1 l of ACN with the aid of ultrasonic bathing and stirred at 75 °C for 30 min under nitrogen. DMAEMA (0.79 g, 5 mmol) was then added dropwise over a period of 30 min. After 10 min, MAA (0.43 g, 5 mmol), PEGMA (0.24 g, 0.5 mmol) and MBA (0.25 g, 1.6 mmol) were added. After 30 min, AIBN (0.13 g, 0.8 mmol) was added as well and 10 min later the temperature was increased to 95–100 °C to start the distillation. The reaction was stopped after collecting 200 ml of distilled ACN and the product was purified with 3 cycles of centrifugation resuspension (5 min × 8000 rpm) and collected as a white powder.

### Synthesis of PMAA@P(MAA-co-PEGMA-co-MBA)@P(DMAEMA-co-MAA-co-PEGMA-co-MBA)@P(MAA-co-PEGMA-co-BAC)

PMAA@P(MAA-co-PEGMA-co-MBA)@P(DMAEMA-co-MAA-co-PEGMA-co-MBA) (0.15 g) were suspended in 750 ml of ACN and stirred at 75 °C for 30 min under nitrogen. MAA (0.39 g, 4.5 mmol), PEGMA (0.11 g, 0.22 mmol) and BAC (0.19 g, 0.72 mmol) were then added. AIBN (0.06 g, 0.4 mmol) was added after 30 min and the reaction medium was stirred for 10 min before increasing the temperature to 95–100 °C and start the distillation. The reaction was stopped after collecting 150 ml of distilled ACN and the product was purified with 3 cycles of centrifugation resuspension (5 min × 5000 rpm) in ACN and collected as a white powder.

### Synthesis of hollow P(MAA-co-PEGMA-co-BAC)@P(DMAEMA-co-MAA-co-PEGMA-co-MBA)@P(MAA-co-PEGMA-co-BAC) NCs

100 mg of PMAA@P(MAA-co-PEGMA-co-MBA)@P(DMAEMA-co-MAA-co-PEGMA-co-MBA)@P(MAA-co-PEGMA-co-BAC) were suspended in a mixture 1:1 of distilled water and ethanol. The suspension was stirred over night at room temperature. The final hollow NCs were isolated and purified by 3 cycles of centrifugation resuspension (5 min × 8000 rpm) in a mixture of water/ethanol (1:1) and collected as a white/transparent powder.

### Loading and release experiments

The loading and release experiments were carried out by using daunorubicin hydrochloride as a model drug. Following a well-established procedure^20,21^, 5 mg of hollow NCs were suspended in 5 ml of phosphate buffer saline (PBS, pH 7.4, 1x) and 5 mg of DNR were then added. The suspension was gently stirred for 72 hours at room temperature. At the end of the process, the non-encapsulated drug was completely removed by centrifugation and resuspension cycles in fresh PBS (5 min × 10.000 rpm × 10 times). The amount of loaded DNR was indirectly determined via UV spectroscopy (Jasco V-650 Spectrophotometer) according to a standard curve (λmax, 484 nm). The concentration of the loaded drug was calculated by the difference in concentration between the original DNR solution and the supernatants collected after the loading process. The parameters used to evaluate the loading were Encapsulation Efficiency (EE% = encapsulated drug (mg)/drug in feeding (mg) × 100) and Loading Capacity (LC% = encapsulated drug (mg)/loaded NCs (mg) × 100).

The release behaviour of the DNR-loaded NCs was investigated in different pH (pH 4, 6 and 7.4), redox (in presence and absence of glutathione) and temperatures (room temperature - 24 ± 2 °C - and 40 °C). Three different buffer solutions were prepared: pH 4 citrate buffer 0.1 M, pH 6 citrate buffer 0.1 M and PBS 1x (pH 7.4). The reducing environments were obtained by adding glutathione to the buffer solutions when needed (final concentration 10 mM). The release profile of DNR was determined with the dialysis bag method. Typically, 0.5 mg of DNR-loaded NCs were suspended and loaded into MWCO 140 kDa dialysis tube and incubated in 30 ml of each buffer solution. At different time points (30 min, 1 h, 2 h, 5 h, 8 h, 10 h, 24 h, 48 h), 1 ml of the solution was withdrawn and analysed. The concentration of each sample was determined with UV spectroscopy by using the standard curve method (λmax, 484 nm).

### *In vitro* cytotoxicity studies

The cytotoxicity of the formulation was evaluated via MTT assay^34^. In particular, MCF-7 (human breast adenocarcinoma cell) and HEK-293 (Human Embryonic kidney 293 cells) cells (0.1 ml, 8 × 10^3^ cells per well) were seeded in 96-well flat-bottomed microplates and let reach 70% confluence in a controlled atmosphere (37 °C, 5% CO_2_ and 95% relative humidity), in order to be used for 24 h treatments. Subsequently, 100 μl of suspension of the appropriate concentrations of free DNR, loaded DNR (0.01–60 µM) or free NCs (0.006–35.2 µg/ml) were added to each well. After an incubation period of 24 h, the treatments were removed and replaced with 100 µl of MTT solution (1 mg/ml diluted in PBS 1x) and the cells were incubated again for 4 h. The solution was then removed and the formed formazan crystals were dissolved in DMSO. The absorbance was measured at 540 nm (reference filter 620 nm) using a microplate reader (Sirio S, SEAC Radim group). The measurements were then converted to percent viability before being presented. Cytotoxicity experiments were repeated three times.

### Bio imaging

Cellular uptake of DNR-loaded NCs was studied with confocal laser scanner microscopy, or CLSM (Leica TCS SP8 MP, inverted confocal microscope with Acousto-Optical Beam Splitter, for the excitation and multiband spectral detector Argon - excitation at 458, 476, 488, 496 & 514 DPSS 561 - excitation at 561 nm (RED). Multiphoton IR laser MaiTai DeepSee from Spectral Physics, excitation @ 780 nm. MCF-7 cells were grown on 22 mm cover slips placed into six-well culture plates (5 * 10^6^) for 24 h in 1.5 mL of culture medium. Subsequently, the cells were treated with free DNR and DNR-loaded NCs (10 µM). After 2 h incubation, the cover slips were recovered and washed twice with PBS, 10% formaldehyde in PBS and PBS again, before placing them onto microscope slides.

### Statistical analysis

The data are shown as the mean ± standard deviation (SD) of the experiments performed at least three times. Statistical comparisons were carried out using Student’s t test. p < 0.05 was considered to be significant.

## Results and Discussion

### Synthesis and characterization of three-stimuli-sensitive NCs

**Figure 1:**
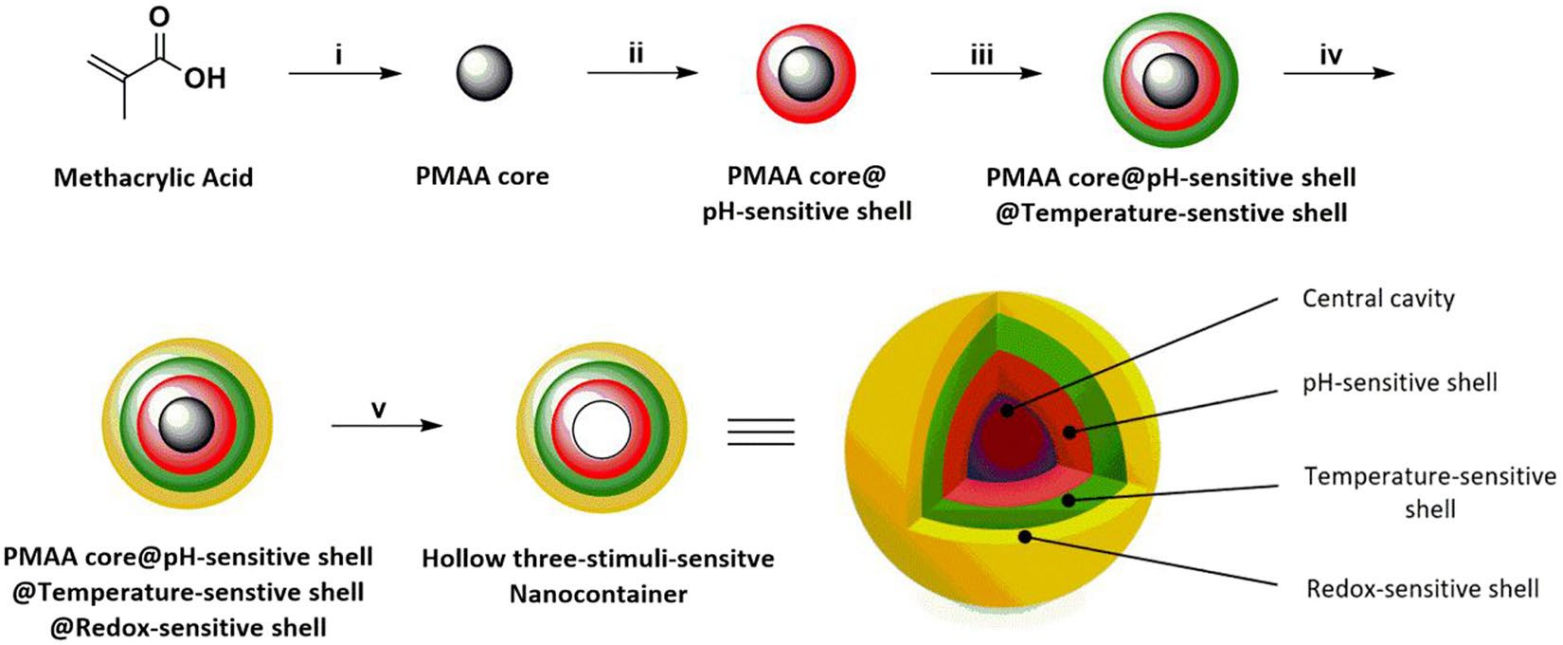
Schematic representation of the synthesis of the three-stimuli-sensitive hollow NCs. (i) DPP: MAA, AIBN, N_2_ bubbling in ACN. (ii) DPP: MAA, PEGMA, MBA, AIBN, N_2_ bubbling in ACN. (iii) DPP: DMAEMA, MAA, PEGMA, MBA AIBN, N_2_ bubbling in ACN. (iv) DPP: MAA, PEGMA, BAC, AIBN, N_2_ bubbling in CAN. (v) Core removal in EtOH/H_2_O dist.

The main objective of this work was the synthesis, characterization and the evaluation of the drug-release properties of three-shell hollow NCs. The system was developed through layer-by-layer method and engineered to release selectively the encapsulated drug under specific tumoral stimuli, like low pH, high temperature and 10 mM GSH concentration, in comparison with physiological conditions. The NCs were fabricated by a five-step process; the first consisted of the core synthesis, followed by the formation of the shells, namely pH-, temperature- and redox-sensitive. As the last step, the core was removed to yield the three-stimuli-sensitive hollow NCs (Fig. 1).

The three shells, each one responsible for a specific sensitivity, and the sacrificial cores were obtained via distillation-precipitation polymerization (DPP), a technique for carrying out free radical chain polymerization used to synthesize spherical and monodisperse particles by taking advantage of the relative insolubility of the oligomers^35^. DPP was chosen because it allowed a facile and straightforward synthesis of monodispersed systems, and its versatility permitted to obtain different sensitivities for the different shells simply by employing different monomers, with specific characteristics.

The first synthesis step was the formation of the monodisperse cores, to be used as sacrificial templates on which the shells were to be built. From a chemical point of view, the formation of the PMAA cores via DPP can be described as a two-sage process. The first stage is named nucleation, where the reaction system is homogenous and the oligomers grow in the continuous phase by the subsequent addition of monomers, until they reach their critical chain length. At that point the oligomers are no longer soluble and precipitate to form the so-called nuclei. These nuclei are unstable and aggregate, forming the mature particles. The resultant suspension of mature particles turns the reaction medium milky white. The second phase is named growth, and involves the capture of residual monomers and oligomers in the medium by the mature PMAA particles through hydrogen-bonding interactions, while the solvent is distilled out of the system. It is worth noticing that only few double bounds are displayed on the surface of the mature particles at the end of the nucleation phase, but the absorption of residual MAA monomers provides the reactive vinyl groups needed for the growth. The absorption of oligomers or their reactions with the adsorbed reactive vinyl groups on the PMAA particles is responsible for the size increase of the particles in this phase^35^.

The syntheses of the shells were achieved via DPP as well. Considering the second-stage DPP to form the pH-sensitive shell, the PMAA cores acted as the mature particles, on the surface of which the monomers and the oligomers (see Supplementary Fig. S1) are absorbed, causing the formation of the cross-linked shell and the consequent increase in size^36^. The principles behind the formation of the temperature- and redox-sensitive shells were identical.

The last step was the removal of the cores, achieved by treating the system with a mixture of water/ethanol, to yield the final hollow NCs.

**Figure 2:**
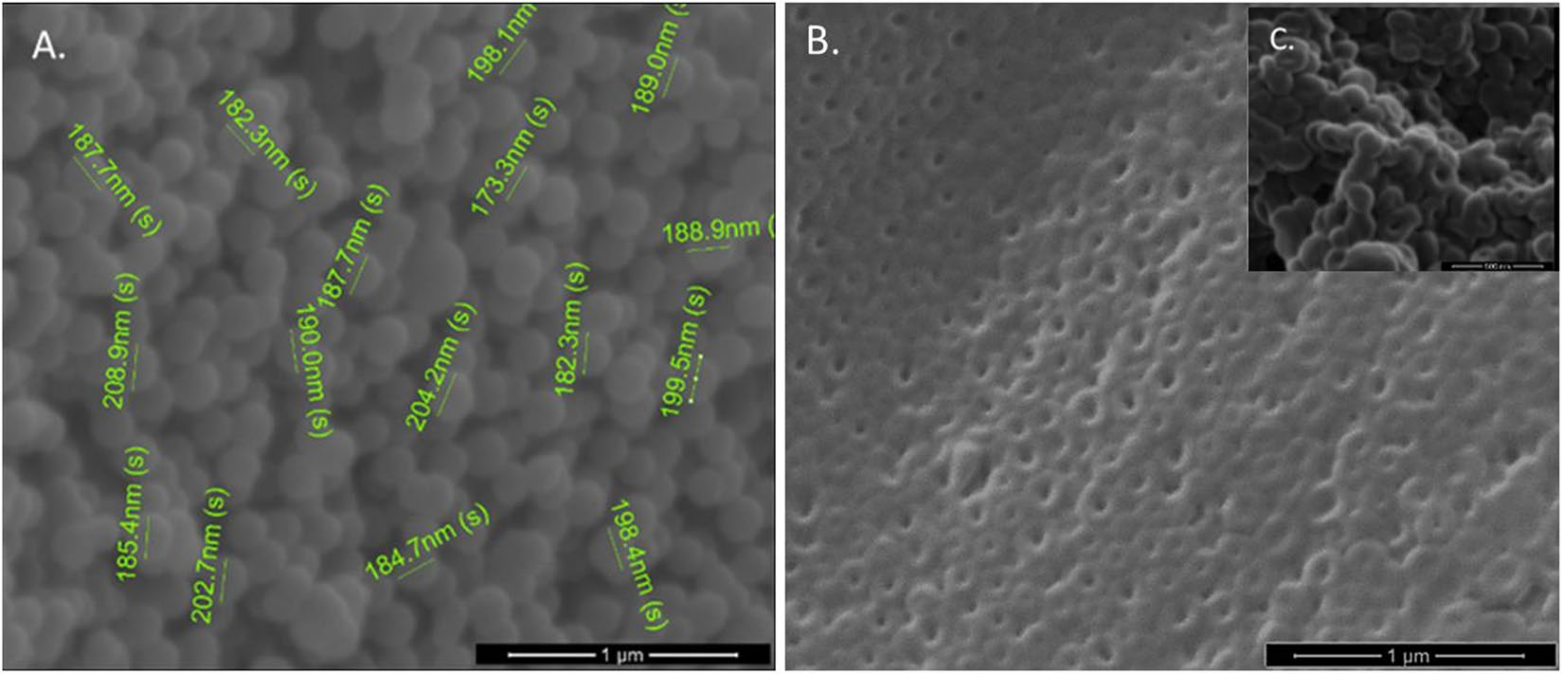
SEM images of the NCs before and after the core removal procedure. (**A**) Three shell/core NPs, (**B**) hollow NCs and (**C**) hollow NCs: detail.

The morphology and the size of the system after each synthesis step were analysed with SEM. The PMAA seeds and the core/shell nanoparticles resulted spherical with rough surfaces, as shown in Fig. 2A for the three shell/core NPs. The shape of the hollow NCs in Fig. 2B,C, with the visible central cavity, confirmed the removal of the sacrificial templates. The SEM images (see Supplementary Fig. S2) and the analysis of the size increments measured with SEM after each layer formation, summarized in Supplementary Table S1, confirms that all the coating procedures were successful. The initial size of the cores was 100 ± 10 nm and each shell increased the size by roughly 30 nm. Specifically, the cores coated with only the first pH-sensitive shell were measured 130 ± 10 nm, 160 ± 15 nm after the synthesis of the second shell and 190 ± 15 nm the three-shell system. No size variation was noticed after the core removal.

Fourier transform infrared (FT-IR) was used to characterize the chemical structure of the system and confirm the synthesis of the shells (see Supplementary Fig. S3). In particular, the PMAA cores were characterized by the peaks at 1701 cm^−1^ (C=O stretching of the carboxylic group), 1487 cm^−1^ (C-H asymmetrical bending vibration) and 1393 cm^−1^,1261 cm^−1^ and 1168 cm^−1^ (C-H symmetrical bending, O-H and C-O bending respectively). The synthesis of the first shell was confirmed by the presence of a new peak at 1531 cm^−1^, attributed to the N-H amide bending (amide II band) of the cross-linking agent MBA. Regarding the second and third shells, their formations could only be confirmed by some variations in intensity of the pre-existing signals, caused by overlaps. In detail, the formation of the second shell was proved by the increase in intensity of the peak at 1261 cm^−1^ (highlighted with the dotted line in Supplementary Fig. S3), due to the overlap between the C-0-C ester stretching of DMAEMA and the O-H bending of MAA. After the third shell synthesis, the intensity of the peak at 1531 cm^−1^ increased because of the overlap of the N-H amide bending peaks belonging to BAC and MBA. The core removal considerably affected the configuration of the spectrum. The peaks of PMAA at 1701 cm^−1^ and 1168 cm^−1^ decreased, uncovering other characteristic peaks, such as the C=O amide stretching, or amide I band, of MBA and BAC at 1640 cm^−1^ and the C-O-C ether asymmetrical stretching at 1110 cm^−1^ of the PEGMA segments.

The pH-dependent variations of the hydrodynamic diameter of the three-stimuli sensitive NCs measured with DLS can be found as Supplementary Table S2. In neutral environment (pH 7) the hydrodynamic diameter of the NCs was 407 nm. Compared to the SEM size of 190 nm, there is a considerable increase. This effect is due to the interaction between the hydrophilic NCs and the aqueous environment, where the NCs assumed a “swollen state”, in contrast to the dry state in SEM samples^37^. When the pH is decreased to 2, the carboxylic groups of PMAA (pKa ca. 5.65) were completely protonated (-COOH) and tended to associate one another via hydrogen bonds. This behaviour caused the formation of aggregates of hollow NCs, which explains the extremely large results of 1840 nm. When the pH was adjusted to 4, the fraction of protonated carboxylic group decreased, but –COOH was still the preponderant form. In this condition the hydrodynamic diameter was found to be 813 nm, which describes a situation where aggregates and single NCs coexist, leading to an intermediate result. When the pH was adjusted to 10, the hydrodynamic diameter of the NCs was found 514 nm. Here, the carboxylic groups of MAA were fully ionized, resulting in the Donnan osmotic swelling of the polymeric network.

### Drug encapsulation profile of three-stimuli-sensitive NCs

To study the drug release profile of the system, the three-stimuli-sensitive NCs were loaded with the anticancer drug DNR, used as a model drug. As summarized in Table 1, EE% and LC% were found to be respectively 85.25 ± 1.36 and 63.03 ± 0.37. The system exhibited excellent encapsulation ability under the employed experimental conditions. These results depend on the capability of the NCs to form electrostatic and hydrogen-bonding interactions with DNR, which are believed to be fundamental to the success of the encapsulation process. In particular, in pH 7.4 the carboxylic groups of PMAA are largely deprotonated and negatively charged, conversely the amino group of the DNR are partially positively charged (pKa 8.6)^38^, allowing the formation of electrostatic interactions. In addition, the PEG chains, the amide moieties of the cross-liking agents and the protonated portion of the carboxylic groups can interact through hydrogen bonding with the functional groups of DNR, such as its carbonyl and hydroxyl groups^39^.

**Table 1:** Results of the drug loading experiments.

EE %	85.26 ± 1.36
LC %	63.03 ± 0.37
mg DNR/ mg NCs	1.71 ± 0.03
µmol DNR/ mg NCs	3.24 ± 0.05

### *In vitro* drug release profile

**Figure 3:**
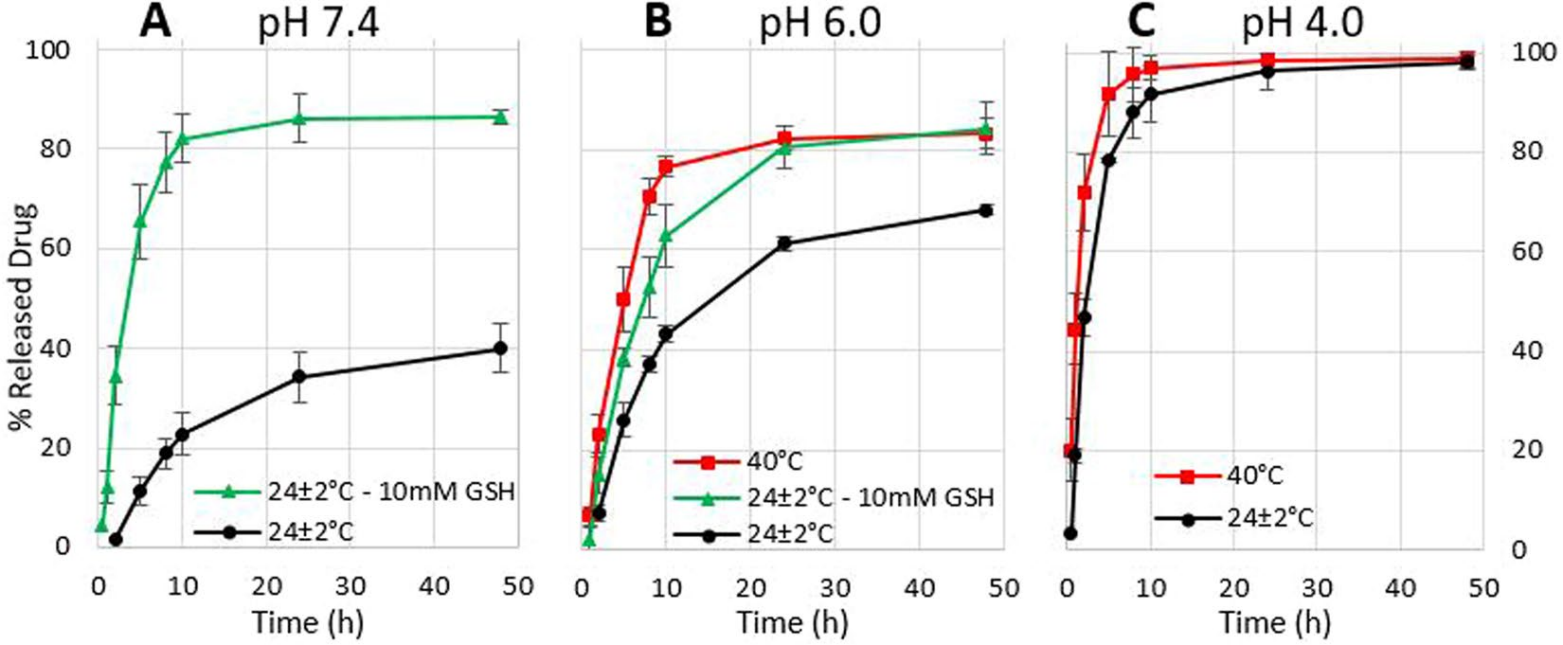
*In vitro* drug release experiments under different conditions. (**A**) Results obtained in pH 7.4; (**B**) results obtained in pH 6.0; (**C**) results obtained in pH 4.0. Error bars are based on mean ± SD of three experiments. Asterisks signify statistically significant difference (p < 0.001) between the treated cells with NCs and the loaded NCs with DNR.

The sensitivities of the hollow NCs were tested by means of *in vitro* drug release experiments under different conditions of pH, temperature and in presence or absence of reducing agents (see Fig. 3). The release behaviour under different pH conditions is shown by the black lines in the graphs. After a treatment of 48 h, the amount of released drug in pH 7.4, 6 and 4 were respectively 40%, 68% and 98%. The effect of pH on the drug release profile could be explained considering the difference in strength of the interaction between the NCs and the DNR molecules, already mentioned for the encapsulation experiments. In particular, in pH 4 the carboxylic groups of PMAA were almost fully protonated, and therefore incapable of forming electrostatic interaction with the positively charged amino groups of DNR. It is also known that hydrogen-bonding interactions are weaker in low pH environments^9^, which contributed to the enhanced release. The difference by 18% after 48 h between the profiles obtained in pH 6 and 7.4 could be justified by considering that the carboxylic groups in pH 6 are partially protonated, unlike in pH 7.4, where they are fully deprotonated and capable of interacting with the DNR molecules to the largest extent. The red lines of Fig. 3 represent the drug release profile of the NCs under the temperature stimuli in different pH. Considering the graph of pH 6 (see Fig. 3B), the amounts of released drug at 24 ± 2 °C and 40 °C after 48 h were respectively 67% and 83%. Interestingly, the release rate was also different. In particular, after 2 h the NCs exposed to 40 °C released 23% of the encapsulated drug, whereas at 24 ± 2 °C only 7%. Furthermore, the system reached 50% of released drug in 5 h at 40 °C and in 10 h at 24 ± 2 °C. Considering pH 4, represented in Fig. 3C, the amounts of released drug after 2 h were 71% at 40 °C and 46% at 24 ± 2 °C, which is an increase by 25%. The temperature sensitivity is due to the polymer PDMAEMA, which exhibits a so-called lower critical solution temperature (LCST). Above this temperature, it becomes insoluble and changes its hydrogen-bonding properties. Specifically, intra- and inter-molecular hydrogen bonds become favoured compared to the interactions with the surrounding environment, such as the solvent and the encapsulated drug^16,30,40^. In this condition, the second shell is no longer available for interacting with DNR, which is consequently released. The change in properties affects the stability of the whole system, explaining the larger amount of released DNR. The last set of drug release experiments was run in presence of glutathione (GSH) as a reducing agent and the trends are showed in green. Regarding pH 7.4 in Fig. 3A, in presence of GSH the system released 87% of DNR after 48 h, which is an increase by 47% compared to pH 7.4 in absence of GSH. In pH 6 with GSH (see Fig. 3B) the system released a larger amount of drug (16% more) than in absence of GSH, respectively 84% and 68%. On the first hand, the cross-linking agent BAC contains a disulphide bridge that can be easily broken in presence of reducing agents. Since BAC was the only cross-linker used for the third layer, in presence of GSH the redox-sensitive shell is quickly broken and removed, causing the part of drug lodged in this shell to be rapidly released. On the other hand, the effect of GSH in pH 7.4 is much more noticeable than in pH 6: it is known that the activity of GSH is pH dependent, and specifically, form pH 7.4 to 6, its activity decreases by 20%^41^. The *in vitro* drug release experiments run in simulated tumour conditions showed the system capable of quickly releasing a larger amount of drug in presence of the three selected stimuli, therefore proving the hollow NCs three-stimuli sensitive.

### Cytotoxicity

**Figure 4:**
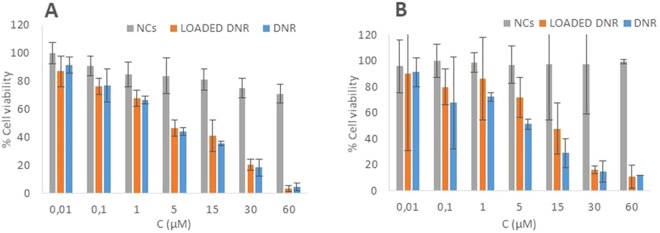
Evaluation of the cell viability via MTT assay. Cytotoxicity in MCF-7 cells (**A**) and HEK-293 (*p = 0,002 < 0.05), (**B**) of empty NCs, DNR-loaded NCs and free DNR. Error bars are based on mean ± SD of three experiments. (*p = 0,001 < 0.05 and the related *on the graph, to show if the differences are relevant).

The cytotoxicity of the DNR-loaded NCs and free NCs was tested with the MTT assay. In particular, MCF-7 human breast carcinoma cells and HEK-293 human embryonic kidney cells were treated for 24 h with free DNR, DNR-loaded NCs (DNR in the range of 0.01–60 µM) and free NCs (0.006–35.2 µg/ml) and the results were expressed as percentages of cell viability. Figure 4 show that the free NCs exerted marginal toxicity, even at high concentrations. The cytotoxicity of free DNR and DNR-loaded NCs was statistically identical independently of the cell line or concentration, confirming that the NCs exclusively acted as carriers capable of releasing almost 100% of the encapsulated drug under the tumour stimuli without affecting its activity.

### Internalization and intracellular localization

**Figure 5:**
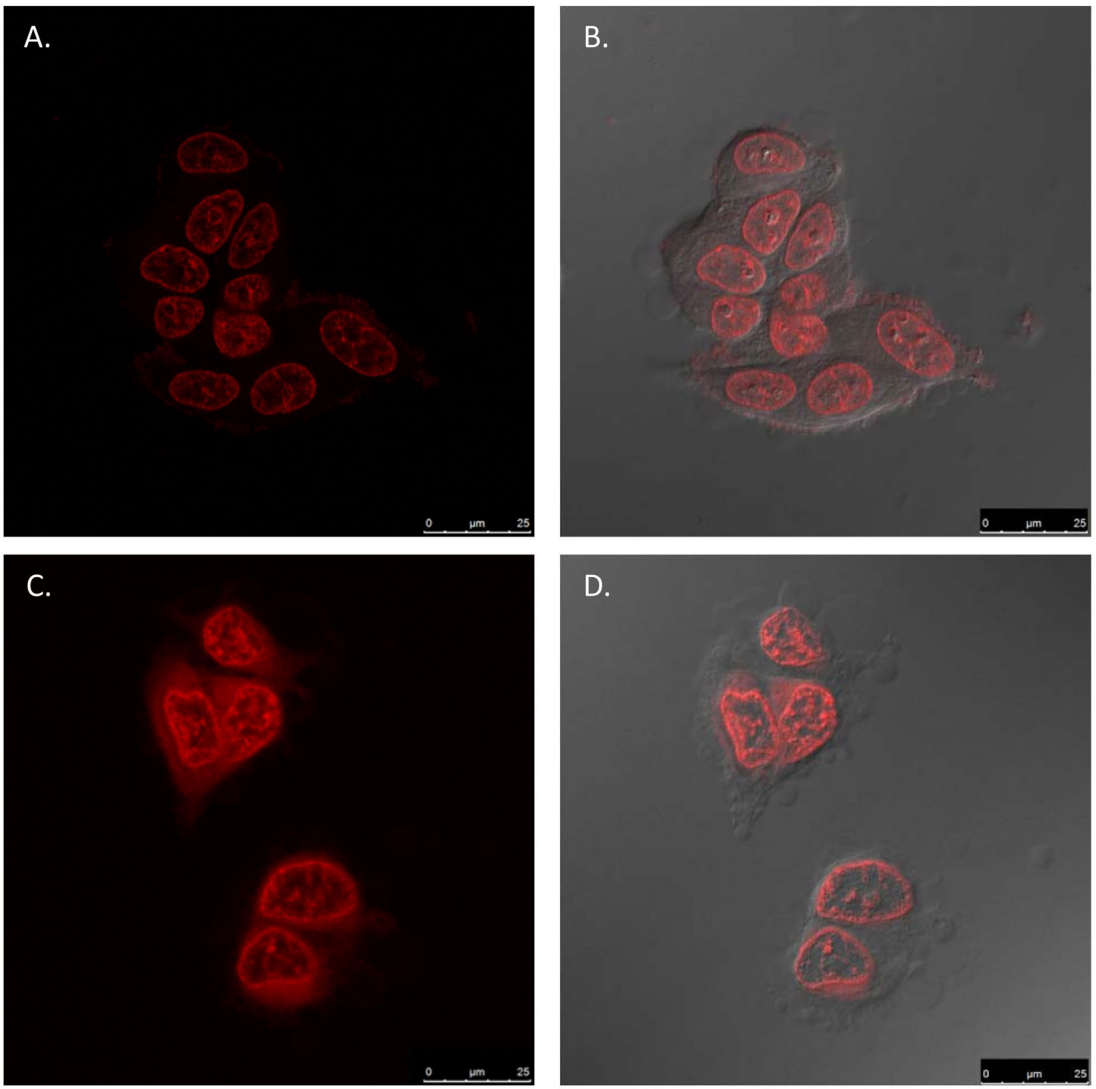
Cellular uptake and intracellular localization of free DNR and DNR-loaded NCs in MCF-7 cells after 2 h incubation. (**A**,**B**) Free DNR and (**C**,**D**) DNR-loaded NCs. (**B** and **D**) Are merged images of optical and fluorescence microscopy.

According to the literature^42–45^, DNR exerts antimitotic and cytotoxic activity through different mechanisms of action. In particular, DNR intercalates the base pairs forming DNR-DNA complexes and inhibits the activity of the enzyme topoisomerase II by stabilizing the DNA-topoisomerase II complex, preventing the enzyme-catalysed reaction. Clearly, the target is DNA and therefore it was crucial to confirm that the encapsulated drug was internalized and localized into the nuclei of the treated cells. Confocal laser scanning microscopy was used to investigate two different phenomena: the cellular uptake and the localization inside the cell of free and encapsulated DNR. As shown in Fig. 5, both free and encapsulated DNR were localized in the nuclei of the cells, where they could exert their functions and stop the cell proliferation. The localization was confirmed with DAPI, used for staining the nuclei. As observed in Supplementary Fig. S4, DAPI (blue), free DNR (red) and NCs@DNR (red) are co-localized in the nuclei.

## Conclusions

The reported drug-loaded NCs can be considered as an ideal formulation, as they showed promising results in terms of sustained drug release and anticancer activity. In particular, the system allowed a sustained release profile of DNR in neutral conditions and burst release in simulated tumour environment, characterized by lower pH, higher temperature and higher concentration of glutathione. In addition, the cytotoxic effect and the intracellular localization of free DNR and the DNR released from the NCs were alike. This formulation affords a platform to develop an injectable controlled release drug delivery system of anthracycline which not only improves the quality of patients’ life but also reduced systematic toxicity and thus the side-effects of chemotherapy.

## Electronic supplementary material

Supplementary Information
