# Supplementary material for: Development of multi-layered and multi-sensitive polymeric nanocontainers for cancer therapy: in vitro evaluation
Source: Sci Rep. 2018 Oct 2;8:14704. doi: 10.1038/s41598-018-32890-5 (PMC6168533; doi:10.1038/s41598-018-32890-5)
Supplement: Supplementary file 1 — Supplementary Information [file 41598_2018_32890_MOESM1_ESM.docx]

# SUPPLEMENTARY INFORMATON

**Development of multi-layered and multi-sensitive polymeric NCs for cancer therapy: in vitro evaluation**

Gianluca Toniolo^1^, Eleni K. Efthimiadou*^1^, George Kordas^1^, Chryssostomos Chatgilialoglu^1,2^

^1^ Institute of Nanoscience and Nanotechnology, NCSR Demokritos, Athens, GREECE

^2^ [Institute for Organic Syntheses and Photoreactivity ISOF](https://www.researchgate.net/institution/Italian_National_Research_Council/department/Institute_for_Organic_Syntheses_and_Photoreactivity_ISOF), [Italian National Research Council](https://www.researchgate.net/institution/Italian_National_Research_Council), Bologna, ITALY

Correspondence and requests for materials should be addressed to Eleni K. Efthimiadou. (email: e.efthimiadou@inn.demokritos.gr)


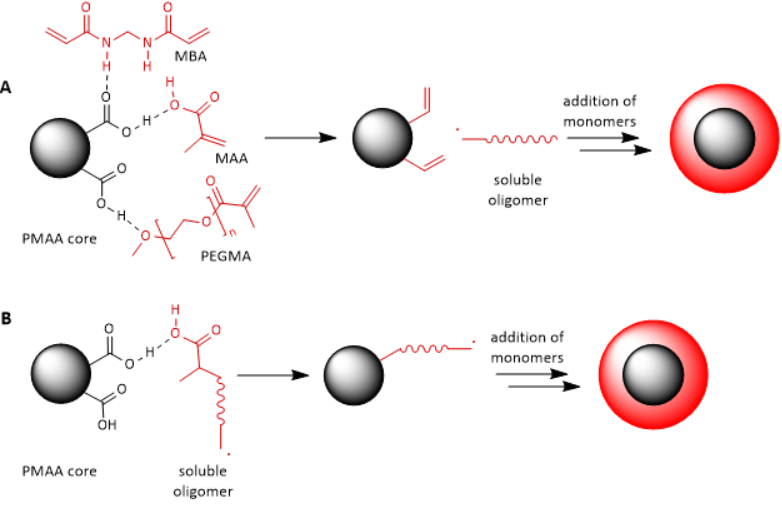


**Supplementary Figure S1. Mechanism of formation of the second shell.** **A**) hydrogen-bonding interactions between the PMAA core and the monomers lead to displaying reactive double bonds on the surface of the cores. The double bonds react with radical oligomers to form the shell. **B**) direct hydrogen-bonding interactions between the PMAA cores and oligomers, which gives reactive PMAA cores surfaces.

**Supplementary Table S1**. **SEM diameters after each synthetic step**

| **Synthetic step** | **Size** |
| --- | --- |
| PMAA cores | 100 ± 10 nm |
| pH-sensitive shell | 130 ± 15 nm |
| Temperature-sensitive shell | 160 ± 15 nm |
| Redox-sensitive shell | 190 ± 15 nm |
| Hollow Nanocontainers | 190 ± 15 nm |

**
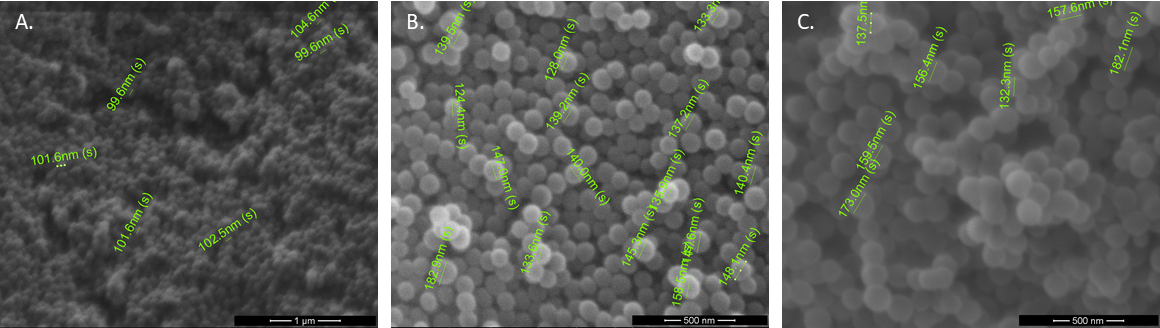
**

**Supplementary Figure S2. SEM images of the NCs before and after the core removal procedure.** **A**) PMAA cores, **B**) pH-sensitive shell/core **C**) two shells/core systems


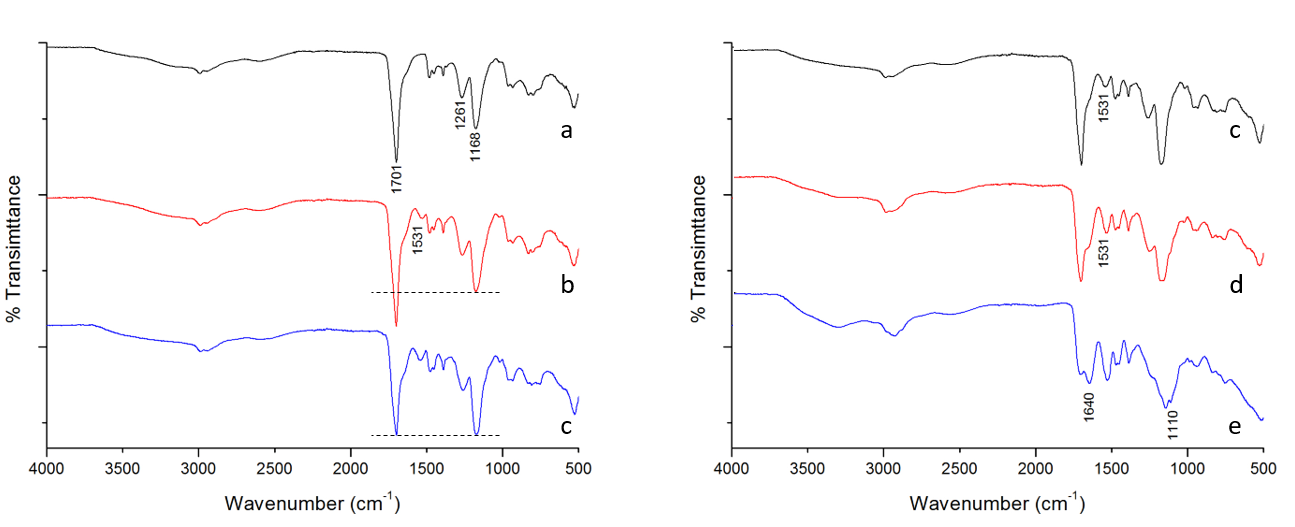

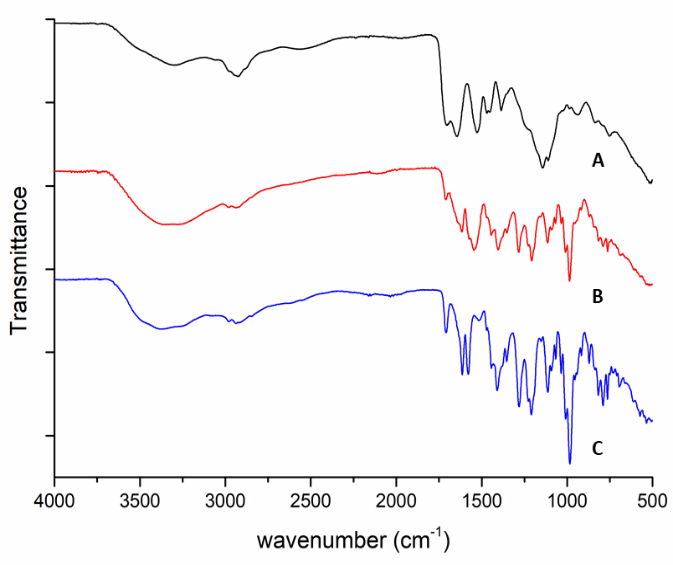

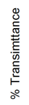

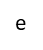

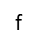

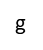


**Supplementary Figure S3.** **FT-IR spectra of the synthesis intermediates and hollow NCs.** **a**) PMAA seeds; **b**) one shell/core; **c**) two shells/core; **d**) three shells/core; **e**) hollow three-shell NCs; **f**) DNR@NCs and **g**) free DNR.

**Supplementary Table S2**: **Hydrodynamic diameter (D_h_) of the three-stimuli sensitive NCs in different pH environment.**

| **pH** | **pH 2** | **pH 4** | **pH 7** | **pH 10** |
| --- | --- | --- | --- | --- |
| **D_h_** | 1840±140.2 nm | 813.8±70.1 nm | 408.6±45.3 nm | 514.0±51.77 nm |
| **PDI** | 0.609±0.085 | 0.625±0.045 | 0.367±0.059 | 0.559±0.122 |


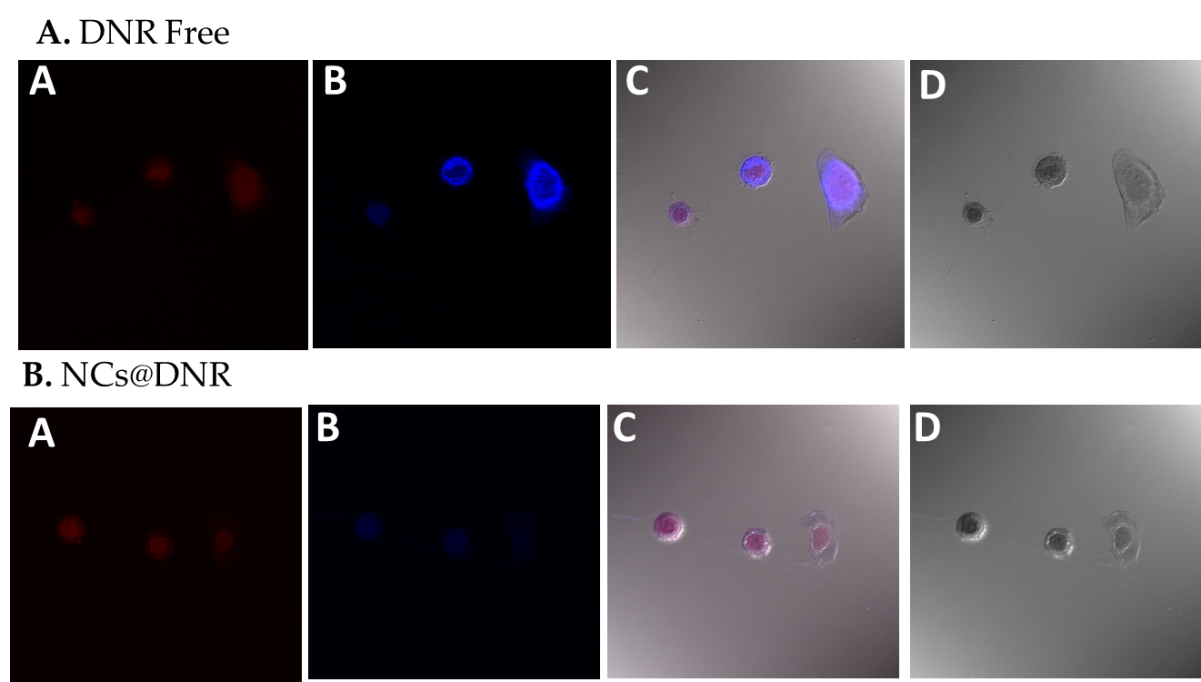


**Supplementary Figure S4. Cellular uptake and intracellular localization of free DNR and DNR-loaded NCs in MCF-7 cells after 2 hours’ treatment, merged with DAPI.** A. Free DNR and B) DNR-loaded NCs: A) DNR, B) DAPI, C) Merged DNR and DAPI, D) Images of the optical microscopy.
